# Supplementary material for: Controls of thermal response of temperate lakes to atmospheric warming
Source: Nat Commun. 2023 Oct 16;14:6503. doi: 10.1038/s41467-023-42262-x (PMC10579293; doi:10.1038/s41467-023-42262-x)
Supplement: Supplementary file 1 — Supplementary Information [file 41467_2023_42262_MOESM1_ESM.pdf]

Supplementary Information for

Controls of thermal response of temperate lakes to atmospheric warming

Jian Zhou, Peter R. Leavitt, Kevin C. Rose, Xiwen Wang, Yibo Zhang, Kun Shi, Boqiang Qin

Content

Supplementary Tables **2**

Supplementary Table 1 The trends of climate variables, water temperatures, and Secchi depth from 1979 to 2017 in 345 lakes. 2

Supplementary Table 2 The distribution of trends in air and water temperatures in stratified (229) and non-stratified (116) lakes. 3

Supplementary Table 3 Frequency distributions of lake geomorphology, land use, climate, water temperatures, and water transparency from the 345 lakes. 4

Supplementary Figures **5**

Supplementary Fig. 1 The distribution of trend differences between water temperatures and air temperature (ETT–ATT: a, c; HTT–ATT: b, d) as a function of epilimnetic total phosphorus and chlorophyll *a* concentrations. 5

Supplementary Fig. 2 The trends in lake stratification depth (a) and strength (b) across 229 stratified lakes. 6

Supplementary Fig. 3 The variations of regional sensible heat flux (*H*) and latent heat flux (*LE*) with air temperature during summer. 7

Supplementary Fig. 4 Distributions of air temperature trend (ATT, a) and epilimnetic temperature trend (ETT, b) with air temperature. 8

Supplementary Tables

Supplementary Table 1 The trends of climate variables, water temperatures, and Secchi depth from 1979 to 2017 in 345 lakes. Summer wind speed, WS; total summer precipitation, TSP; Summer humidity, humidity; summer shortwave radiation, SR; summer longwave radiation, LR; regional summer sensible heat flux (*H*); regional summer latent heat flux (*LE*); summer air temperatures, SuAT; spring air temperature, SpAT; fall air temperature, FaAT; winter air temperature, WiAT; Epilimnetic temperature trend, ETT; Hypolimnetic temperature trend, HTT; Secchi depth, Secchi.

	Mean	Minimum	Median	Maximum
WS (m s^{-1} $decade^{-1}$)	−0.04	−0.22	−0.05	0.13
TSP (mm $decade^{-1}$)	−1.8	−90.4	−4.8	85.7
Humidity (g m^{-3} $decade^{-1}$)	0.13	−0.83	0.16	0.96
SR (W m^{-2} $decade^{-1}$)	1.7	−11.8	1.9	22.3
LR (W m^{-2} $decade^{-1}$)	1.7	−7.3	1.8	7.1
H (W m^{-2} $decade^{-1}$)	−1.8	−17.7	−1.3	11.6
LE (W m^{-2} $decade^{-1}$)	−0.1	−12.7	−0.6	15.3
SuAT (°C $decade^{-1}$)	0.36	−0.90	0.32	1.99
SpAT (°C $decade^{-1}$)	0.45	−0.77	0.40	2.07
FaAT (°C $decade^{-1}$)	0.56	−0.78	0.48	2.77
WiAT (°C $decade^{-1}$)	−0.10	−2.18	0.03	1.79
ETT (°C $decade^{-1}$)	0.44	−1.63	0.43	2.64
HTT (°C $decade^{-1}$)	−0.12	−2.05	−0.05	1.23
Secchi (m $decade^{-1}$)	−0.03	−3.08	0.02	2.06

Supplementary Table 2 The distribution of trends in air and water temperatures in stratified (229) and non-stratified (116) lakes. ETT–ATT, the difference between epilimnetic temperature trend (ETT) and air temperature trend (ATT) in summer; HTT–ATT, the difference between hypolimnetic temperature trend (HTT) and ATT in summer; ETT–HTT, the difference between ETT and HTT in summer.

	Stratified lakes (229 lakes)	Non-stratified lakes (116 lakes)
ATT (°C $decade^{-1}$)	0.35 ± 0.26	0.38 ± 0.43
ETT (°C $decade^{-1}$)	0.46 ± 0.49	0.39 ± 0.70
HTT (°C $decade^{-1}$)	−0.12 ± 0.47	/
ETT–ATT (°C $decade^{-1}$)	0.11 ± 0.48	0.006 ± 0.59
HTT–ATT (°C $decade^{-1}$)	−0.47 ± 0.53	/
ETT–HTT (°C $decade^{-1}$)	0.59 ± 0.62	/

Supplementary Table 3 Frequency distributions of lake geomorphology, land use, climate, water temperatures, and water transparency from the 345 lakes. Lake geomorphic characteristics included lake area, maximum depth, area/depth ratio, volume, water residence time, elevation, and watershed area. Land use was characterized into agriculture, developed, water, forest, wetlands, grass, and shrubland. Climatic variables included WS, TSP, humidity, SR, LR, *H*, *LE*, SuAT, SpAT, FaAT, and WiAT. Water temperature (T) were characterized into epilimnion and hypolimnion. Water transparency was categorized by Secchi depth.

	Mean	Minimum	Median	Maximum
Lake area (km^2)	21.1	0.1	1.3	1141.3
Maximum depth (m)	23.3	1.5	15.2	370
Area/depth ratio ((km^2 m^{-1})	0.64	0.004	0.1	31.7
Volume (mcm)	570.2	0.3	6.8	59096.6
Water residence time (days)	777.1	1.9	343.4	14301.3
Elevation (m)	242.5	0	247	1657
Watershed area (km^2)	1827.5	0.3	23.0	194674.4
Agriculture (%)	11.7	0	2.9	81.9
Development (%)	13.9	0	4.8	97.8
Water (%)	4.9	0	2.3	50.8
Forest (%)	53.0	0	64.0	96.2
Wetland (%)	3.8	0	1.8	27.0
Grass (%)	5.5	0	0.8	65.2
Shrubland (%)	1.7	0	0.91	28.9
WS (m s^{-1})	2.76	1.11	2.72	4.95
TSP (cm)	18.7	1.5	18.4	56.5
Humidity (g m^{-3})	12.8	5.5	12.8	20.4
SR (W m^{-2})	202.8	157.2	200.1	274.5
LR (W m^{-2})	362.1	310.6	359.9	414.6
H (W m^{-2})	−107.0	−150.4	−107.2	−42.5
LE (W m^{-2})	−28.5	−131.0	−27.1	−6.6
SuAT (°C)	20.6	13.1	20.4	27.4
SpAT (°C)	10.6	5.1	9.6	23.7
FaAT (°C)	6.5	−0.2	6.0	22.0
WiAT (°C)	−4.7	−14.2	−6.2	16.8
Epilimnetic T (°C)	22.0	11.8	22.5	29.7
Hypolimnetic T (°C)	9.6	4.8	9.0	19.8
Secchi depth (m)	4.0	0.4	3.5	16.0

Supplementary Figures

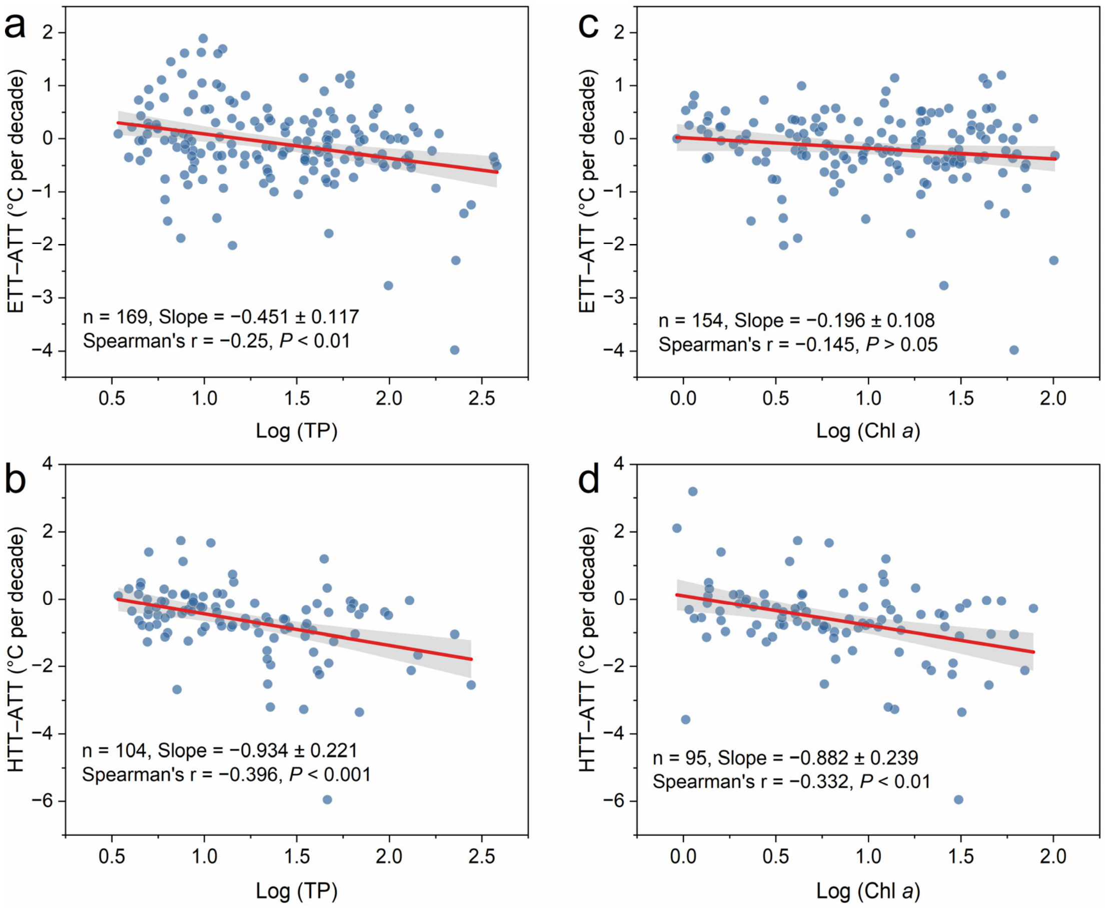

Supplementary Fig. 1 The distribution of trend differences between water temperatures and air temperature (ETT–ATT: a, c; HTT–ATT: b, d) as a function of epilimnetic total phosphorus (TP) and chlorophyll *a* (Chl *a*) concentrations (μg L^{-1}). ETT–ATT, the difference between epilimnetic temperature trend (ETT) and air temperature trend (ATT); HTT–ATT, the difference between hypolimnetic temperature trend (HTT) and ATT. Pairwise correlations were examined by Spearman's correlation coefficient. The data of TP and Chl *a* were log-transformed.

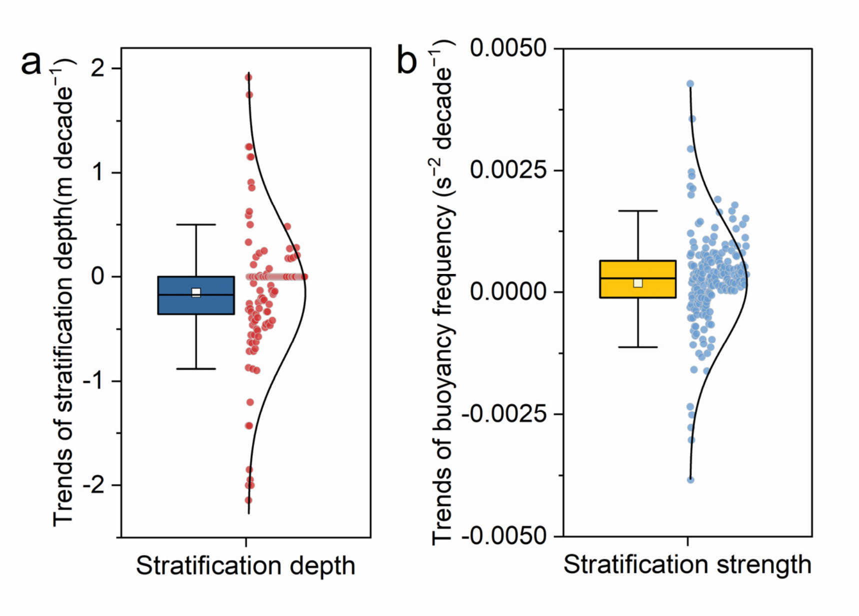

Supplementary Fig. 2 The trends in lake stratification depth (a) and strength (b) across 229 stratified lakes. Stratification depth was defined as depths down to and including the upper boundary of the metalimnion. Stratification strength was represented using buoyancy frequency.

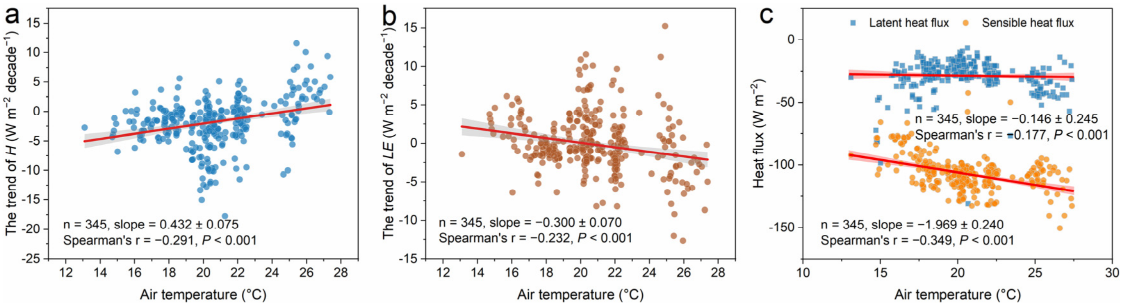

Supplementary Fig. 3 The variations of regional sensible heat flux (*H*) and latent heat flux (*LE*) with air temperature during summer. a, b The trends of *H* and *LE* distributed with air temperature. **c** The values of *H* and *LE* varied with air temperature.

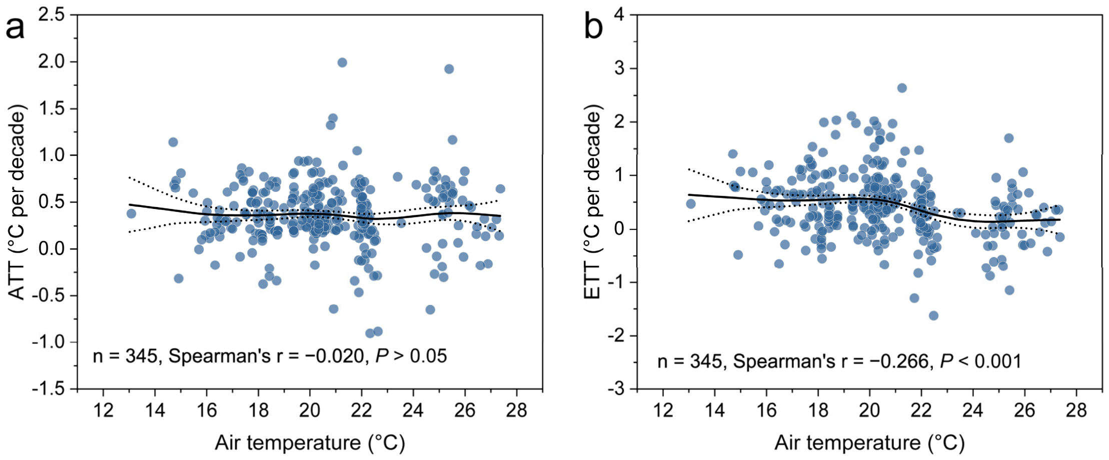

Supplementary Fig. 4 Distributions of air temperature trend (ATT, a) and epilimnetic temperature trend (ETT, b) with air temperature. The black line is a generalized additive model fit to the data points, whereas the dotted line represents the pointwise 95% credible interval of the fitted values. Pairwise correlations between ATT, ETT, and air temperature were examined by Spearman's correlation coefficient.
